# Serious mistakes in meta-analysis of homeopathic research

**Published:** 2017

**Authors:** G Vithoulkas

**Affiliations:** * International Academy of Classical Homeopathy, Alonissos, Greece

**Keywords:** serious mistakes, meta-analysis, homeopathic research

## Abstract

The article discussed the immanent problems of meta-analyses selecting a number of independent trials in homeopathy, within which, the purpose was to examine the effectiveness of homeopathic treatment. Our focus lied in clarifying that the complex effects of homeopathic treatment known from history and day-to-day practice have not been respected so far.

The examination of most of the homeopathic trials showed that studies rarely account for homeopathic principles, in order to assess the effectiveness of the treatment. The main flaw was that trials reflect the point of view that the treatment with a specific remedy could be administered in a particular disease. However, homeopathy aims to treat the whole person, rather than the diseases and each case has to be treated individually with an individualized remedy. Furthermore, the commonly known events during the course of homeopathic treatment, such as “initial aggravation” and “symptom-shift” were not considered in almost all the studies. Thus, only few trials were eligible for meta-analyses, if at all. These and other factors were discussed and certain homeopathic principles were suggested to be respected in further trials. It is expected, that a better understanding of homeopathic principles would provide guidelines for homeopathic research, which are more acceptable to both homeopathy and conventional medicine.

In the field of homeopathy, meta-analyses as well as randomized controlled trials face the conflict between fulfilling statistical demands and meeting the homeopathic reality. Due to substantial flaws, the outcome of former meta-analyses of placebo‐controlled RCTs on the use of homeopathy [**1**-**5**] are inconclusive. Explanations for this have been a different and sometimes arbitrary selection of trials [**6**], a suspected publication bias [**2**], heterogeneity [**1**] and a low quality of the existing trials [**2**,**3**,**5**,**7**]. However, we wanted to focus on an aspect that was not sufficiently stressed in the past, but was neither of less importance: The fact that most trials included in the analyses did not respect either the homeopathic principles or the indications of the prescribed homeopathic remedies. Recently, a tool for the quality-assessment of homeopathic trials was introduced into the research debates [**8**] and meta-analytic parameters. It resulted again in a minimized set of trials to be analyzed. Further, the tool has not been evaluated independently so far. To our concern, it still does not allow a differentiated and accurate appraisal of the conducted trials. The following comments should help clarifying many of the inherent issues of homeopathic methodology that are causing, and may continue to cause, confusing results.

For example, homeopathy demands individual assessment of each case in order to reveal the remedy that will have the best possible therapeutic effect on the individual patient (Law of similarity). However, in almost all the trials reviewed in the meta-analyses, this critical parameter was clearly ignored. Further, in homeopathy, a sound knowledge of the properties of the remedies is of great importance. This is a fact that again seems to be neglected by most of the researchers. As an example, we wanted to comment on the study of Rhus-tox D6, which was tested in osteoarthritis and found to have no effect [**9**]. Not only, that the law of similarity was not respected, but also deciding the remedy based on the pathology was wrong. As it is commonly known to homeopaths, Rhus–tox is almost never indicated in osteoarthritis cases, although it has been useful in some cases of fibrositis and some rheumatic diseases. Other remedies like Causticum, the Kali salts, the Calcarea salts or the Natrium salts could have been tried for this pathology under a specific protocol, but Rhus-tox should have been excluded. The negative conclusion reached by this study would be similar to testing, for example, the use of antibiotics in treating anxiety neurosis, finding that they do not work in this pathology, and then concluding that all conventional medicine is useless! 

Keeping this in mind, this and all similar trials are seriously flawed from the homeopathic point of view. Not even one quarter of the existing studies tested individualized homeopathy [**6**] and all the trials in the Lancet meta-analysis [**1**] exhibit the problem of using a remedy for a specific pathology. Hence, the overall conclusions are compromised. This means that all the research work and expenditure put in such trials added little to the understanding of the effectiveness of homeopathy as a complementary therapeutic method. 

The most recent meta-analysis respected the fact that the individualized method and a homeopathic quality-assessment are essential for the purpose of a fair evaluation of the effectiveness of homeopathic interventions. Still, only 19 out of the 32 placebo-controlled randomized controlled trials were found to have acceptable “model validity” [**10**]. Most of those trials that investigated acute conditions or very advanced stages of pathology were that the effect of homeopathy was more comparable to the conventional understanding of the improvement [**11**]. In most other chronic diseases, the individual undergoes an “initial aggravation of the existing symptoms” or a “symptom shift” [**11**]. Generally, it appeared that the proposed “model validity” could be working only for the cases in which the first intervention with a remedy would have shown some beneficial effect for the patient. The contemporary state of health of the western population, especially the European and North American patients, concerning their chronic conditions, require a treatment of few years and will need a series of remedies [**12**], before they showed tangible therapeutic results. The reason is that the immune system in the majority of such cases is very compromised [**11**]. This aspect was not considered in the rating process. Also, patients in the beginning stages of chronic diseases might even undergo such severe initial aggravation, after a correct prescription, that they drop out from the studies or interfere with allopathic drugs in order to minimize the intensity of the aggravated symptoms. In both cases, the evaluation would be misleading. The apparent initial aggravation is, from the homeopathic point of view, considered as a positive sign and a re-awakening of the immune system of the patient. This issue has not been dealt with at all in homeopathic research so far nor have the initial aggravations been taken into consideration in the planning of homeopathic trials. Thus, it further contributes to the reduced amount of suitable trials for meta-analysis.

Therefore, we wanted to emphasize that the homeopathic community needs a standardized protocol [**13**] and should not accept research that does not comply with, or does not respect the homeopathic principles. 

**Which are these homeopathic principles to be respected?**

1. Homeopathy does not treat diseases, but only diseased individuals. Therefore, every case may need a different remedy although the individuals may be suffering from the same pathology. This rule was violated by almost all the trials in most meta-analyses.

2. In the homeopathic treatment of serious chronic pathology, if the remedy is correct usually a strong initial aggravation takes place [**14**-**16**]. Such an aggravation may last from a few hours to a few weeks and even then we may have a syndrome-shift and not the therapeutic results expected. If the measurements take place in the aggravation period, the outcome will be classified negative. 

This factor was also ignored in most trials [**10**]. At least sufficient time should be given in the design of the trial, in order to account for the aggravation period. The contrary happened in a recent study [**17**], where the aggravation period was evaluated as a negative sign and the homeopathic group was pronounced worse than the placebo [**18**].

3. In severe chronic conditions, the homeopath may need to correctly prescribe a series of remedies before the improvement is apparent. Such a second or third prescription should take place only after evaluating the effects of the previous remedies [**11**]. Again, this rule has also been ignored in most studies. 

4. As the prognosis of a chronic condition and the length of time after which any amelioration set in may differ from one to another case [**11**], the treatment and the study-design respectively should take into consideration the length of time the disease was active and also the severity of the case. 

5. In our experience, Homeopathy has its best results in the beginning stages of chronic diseases, where it might be possible to prevent the further development of the chronic state and this is its most important contribution. Examples of pathologies to be included in such RCTs trials are ulcerative colitis, sinusitis, asthma, allergic conditions, eczema, gangrene rheumatoid arthritis as long as they are within the first six months of their appearance.

## Conclusion

In conclusion, three points should be taken into consideration relating to trials that attempt to evaluate the effectiveness of homeopathy. 

First, it is imperative that from the point of view of homeopathy, the above-mentioned principles should be discussed with expert homeopaths before researchers undertake the design of any homeopathic protocol. 

Second, it would be helpful if medical journals invited more knowledgeable peer-reviewers who understand the principles of homeopathy. 

Third, there is a need for at least one standardized protocol for clinical trials that will respect not only the state-of-the art parameters from conventional medicine but also the homeopathic principles [**13**].

Fourth, experience so far has shown that the therapeutic results in homeopathy vary according to the expertise of the practitioner. Therefore, if the objective is to validate the homeopathic therapeutic modality, the organizers of the trial have to pick the best possible prescribers existing in the field. 

Only when these points are transposed and put into practice, the trials will be respected and accepted by both homeopathic practitioners and conventional medicine and can be eligible for meta-analysis.
